# N-Acetylglucosamine Functions in Cell Signaling

**DOI:** 10.6064/2012/489208

**Published:** 2012-12-05

**Authors:** James B. Konopka

**Affiliations:** Department of Molecular Genetics and Microbiology, Stony Brook University, Stony Brook, NY 11794-5222, USA

## Abstract

The amino sugar N-acetylglucosamine (GlcNAc) is well known for the important structural roles that it plays at the cell surface. It is a key component of bacterial cell wall peptidoglycan, fungal cell wall chitin, and the extracellular matrix of animal cells. Interestingly, recent studies have also identified new roles for GlcNAc in cell signaling. For example, GlcNAc stimulates the human fungal pathogen *Candida albicans* to undergo changes in morphogenesis and expression of virulence genes. Pathogenic *E. coli* responds to GlcNAc by altering the expression of fimbriae and CURLI fibers that promote biofilm formation and GlcNAc stimulates soil bacteria to undergo changes in morphogenesis and production of antibiotics. Studies with animal cells have revealed that GlcNAc influences cell signaling through the posttranslational modification of proteins by glycosylation. O-linked attachment of GlcNAc to Ser and Thr residues regulates a variety of intracellular proteins, including transcription factors such as NF**κ**B, c-myc, and p53. In addition, the specificity of Notch family receptors for different ligands is altered by GlcNAc attachment to fucose residues in the extracellular domain. GlcNAc also impacts signal transduction by altering the degree of branching of N-linked glycans, which influences cell surface signaling proteins. These emerging roles of GlcNAc as an activator and mediator of cellular signaling in fungi, animals, and bacteria will be the focus of this paper.

## 1. Introduction

 Recent studies are revealing new ways in which GlcNAc directly and indirectly mediates cell signaling. These findings raise the questions of what are the sources of GlcNAc that induce cell signaling and how does GlcNAc affect cell function? Therefore, the first section of this paper will summarize the diverse types of GlcNAc polymers at the cell surface, as turnover of these polymers is a likely source of GlcNAc that can induce signaling. These polymers include bacterial cell wall peptidoglycan, fungal cell wall chitin, and the extracellular matrix of animal cells. The mechanisms by which GlcNAc stimulates cell signaling will then be reviewed in subsequent sections with examples from fungal, mammalian, and prokaryotic cells. The final section will examine the possibility of interspecies communication mediated by GlcNAc, which seems likely based on the ubiquitous nature of this amino sugar.

The ability of GlcNAc to induce responses in fungi will be examined first, since recent studies have identified the first eukaryotic GlcNAc transporter and other studies have shown that GlcNAc can act directly as a signal inducer [1, 2]. In particular, GlcNAc stimulates the human fungal pathogen *Candida albicans* to induce the expression of virulence genes and a shift from growing as unicellular budding yeasts to instead forming multicellular filamentous hyphal cells [3]. Subsequent comparison of these responses to the GlcNAc-induced changes in bacteria will reveal distinct ways for GlcNAc to activate signaling, including the direct interaction of GlcNAc with transcriptional regulators that influence production of virulence factors [4, 5].

Another role for GlcNAc as a modifier of signaling has been identified in animal cells in which GlcNAc influences protein glycosylation. This includes three distinct types of glycosylation that all have the capacity to modulate cell-signaling pathways. One is the O-linked attachment of GlcNAc to Ser and Thr residues as a form of posttranslational modification, which occurs on a large number of cytosolic and nuclear proteins, including those that mediate signaling [6]. Another is the ability of GlcNAc to increase branching of N-linked glycans attached to cell surface proteins, which can alter their stability or signaling activity [7]. A third mechanism is the attachment of GlcNAc onto fucose residues on the Notch family of receptors, which alters their ligand specificity [8]. One underlying aspect of all three of these forms of glycosylation is that they appear to be regulated by the levels of their substrate, UDP-GlcNAc. They are therefore thought to represent a mechanism to signal nutritional effects, since UDP-GlcNAc synthesis is regulated by levels of hexose sugars, Acetyl CoA, amino acids, and nucleotides [9]. 

Another fundamental issue that all organisms face is the challenge of regulating the fate of GlcNAc in the cell. Cells must coordinate exogenous GlcNAc taken up by the cell with the de novo synthesis of GlcNAc, and also whether GlcNAc is used in the catabolic pathways that break down GlcNAc for nutrition or shunted into the anabolic pathways that create UDP-GlcNAc for use protein modification and formation of cell surface structures. This is critical as imbalances in GlcNAc metabolism are deleterious to cell growth [2, 10, 11].

## 2. GlcNAc Roles in Cell Surface Structure

### 2.1. GlcNAc Functions at the Fungal Cell Surface

GlcNAc plays several important roles at the cell surface of fungi. One critical role is in the formation of the cell wall, the innermost layer of which is composed of chitin, a polymer of *β*-(1,4) linked GlcNAc. GlcNAc is also important for modification of cell-surface proteins. It forms part of the N-linked polysaccharide chain that is added to glycosylated proteins and is a building block in the synthesis of GPI-anchors that maintain certain proteins in the plasma membrane. UDP-GlcNAc is the substrate (donor sugar) for synthesis of chitin, N-linked glycans, and GPI anchors, making it an important metabolite whose synthesis is tightly controlled. N-linked glycosylation and GPI anchors are not likely to be sources of exogenous GlcNAc for signaling, since N-linked glycans are relatively stable at the cell surface and GlcNAc is deacetylated during the synthesis of GPI anchors. In contrast, chitin is rapidly turned over during the cell wall remodeling that occurs to allow for the expansion of new cell growth. Thus, chitin breakdown is the most likely source of GlcNAc that will then be available for cell signaling. As will be described further in a later section, this exogenous GlcNAc provides opportunities for intra- and interspecies communication.

### 2.2. GlcNAc Roles in the Extracellular Matrix of Animal Cells

GlcNAc is also expected to be abundant at the surface of multicellular organisms. As described above for fungi, GlcNAc is added to proteins during N-linked glycosylation and it is an intermediate in the synthesis of GPI-anchors. However, the extracellular matrix of animal cells also contains a variety of different sugar polymers, many of which contain GlcNAc [12]. For example, hyaluranon is a polymer of GlcNAc and glucaronic acid. The extensive layer of sugar polymers in the extracellular matrix undergoes remodeling that could liberate significant amounts of exogenous GlcNAc for cell signaling.

### 2.3. Roles of GlcNAc in Bacterial Cell Surface Structure

GlcNAc plays a variety of roles in cell surface structure of bacteria analogous to its roles in eukaryotic cells, although there are major differences. GlcNAc is important in the cell wall of both Gram-positive and Gram-negative bacteria, and it also forms part of the cell wall of archaea. In particular, GlcNAc is a major component of cell wall peptidoglycan, which is comprised of a sugar backbone containing alternating residues of *β*-(1,4) linked GlcNAc and *N*-acetylmuramic acid [13]. A peptide chain of three to five amino acids attached to the *N*-acetylmuramic acid is cross-linked to the peptide chain of another strand to create a rigid wall. The presence of GlcNAc in peptidoglycan is of particular interest because of the large amount of turnover of this cell wall layer during growth, which releases significant amounts of GlcNAc [13]. The GlcNAc liberated during cell wall remodeling can induce cell signaling, and there are also regulatory mechanisms that are in place to coordinate the recycling of GlcNAc with the de novo synthesis of this sugar to create UDP-GlcNAc. The release of GlcNAc during cell wall remodeling also provides opportunities for interspecies interactions, as will be described below. 

UDP-GlcNAc is also a precursor for the synthesis of the lipid A of lipopolysaccharide (LPS) that is the major component of the outer membrane of Gram-negative bacteria. LPS is also well known as an important inducer of inflammation in humans. Synthesis of lipid A involves the conversion of UDP-GlcNAc to a phosphorylated glucosamine disaccharide with multiple fatty acids attached [14]. LPS appears to be stable in the outer membrane and is not subject to cycles of degradation and recycling, as is peptidoglycan. In Gram-positive bacteria GlcNAc is also found in teichoic acids, which are a component of the cell wall [15].

A distinct type of GlcNAc polymer, poly-*β*-(1,6) N-acetyl-D-glucosamine (PNAG), is also present at the cell surface and has been found to be important for biofilm formation in a wide range of bacteria. In *E. coli, *PNAG produced by PgaC is then partially de-*N*-acetylated by PgaB [16]. Partially de-*N*-acetylated PNAG is thought to be the functionally relevant form, as deletion of *pgaB *results in the retention of the fully acetylated polymer in the periplasm and abolishment of biofilm formation [16, 17]. There are no reports to indicate whether GlcNAc regulates production or recycling of PNAG.

## 3. GlcNAc Functions in *C. albicans* and Other Fungi

### 3.1. Overview of GlcNAc Signaling in Fungi

Addition of GlcNAc to the extracellular medium has been shown to activate signal transduction pathways in a variety of yeasts that promote changes in morphogenesis and gene regulation. For example, GlcNAc induces *C. albicans *[18], *Candida lusitaniae *[19], and *Yarrowia lipolytica *[20] to switch from growing as budding yeast cells to forming long filamentous hyphal or pseudohyphal cells. GlcNAc also stimulates a type of epigenetic switch that causes some *Candida* species that are homozygous at the mating type loci to grow in a different cellular morphology in a process known as White-Opaque switching [21]. GlcNAc induces specific patterns of gene expression in hyphal cells and the White-Opaque cells, most of which are probably induced indirectly as part of the hyphal or White-Opaque regulatory programs. However, GlcNAc also appears to directly induce the expression of the genes that are needed for its catabolism [22].

GlcNAc signaling has been best studied in the human fungal pathogen *C. albicans*, as the more commonly studied model yeasts *Saccharomyces cerevisiae* and *Schizzosaccharomyces pombe* lack the genes required to catabolize GlcNAc and do not appear to show a phenotypic response to this sugar. Genetic analysis of *C. albicans* indicates that GlcNAc activates at least two pathways. One pathway appears to activate cAMP signaling, which then triggers hyphal morphogenesis and expression of virulence factors [22–24]. GlcNAc activation of cAMP signaling also induces White-Opaque switching [21]. The second pathway activated by GlcNAc, which is independent of cAMP signaling, results in increased expression of the genes needed to catabolize GlcNAc [22, 25, 26].

### 3.2. *C. albicans* Senses Intracellular GlcNAc

The initial studies that attempted to determine whether GlcNAc has to be taken up by *C. albicans *to induce hyphal morphogenesis led to conflicting conclusions [18, 27]. A serious limitation of these earlier studies was the lack of genetic approaches available at the time to study this diploid organism. However, the more recent discovery of a GlcNAc transporter (Ngt1) in *C. albicans *has permitted new approaches that have provided important new insights into GlcNAc signaling [1]. Ngt1 was first identified in a proteomic analysis of plasma membrane proteins from cells that were induced by GlcNAc to form hyphae. The presence of ~12 transmembrane domains in Ngt1 suggested similarity to the major facilitator superfamily of transporters. This prediction was verified in part by showing that an *ngt1Δ* deletion mutant was very strongly impaired in GlcNAc uptake. Further proof was obtained by showing that heterologous expression of *NGT1* in *S. cerevisiae* conferred the ability to take up GlcNAc. These experiments took advantage of the fact that *S. cerevisiae* lacks an *NGT1* ortholog and does not take up GlcNAc efficiently. Additional studies showed that Ngt1 was specific for GlcNAc; other related sugars did not compete for the uptake of ^3^H-GlcNAc by Ngt1 [1]. Since *NGT1* is the first eukaryotic GlcNAc transporter gene to be identified, it has also turned out to be a useful new tool for studying the roles of GlcNAc in other organisms, such as allowing GlcNAc analogs to be taken up by *S. cerevisiae* [28]. Also, homologous genes can now be identified in other organisms and targeted for study. For example, a full-genome RNAi study in *Caenorhabditis elegans* found that decreased expression of an *NGT1* homolog caused defects in early embryogenesis [29].

The phenotypes of the *C. albicans ngt1Δ* cells provided support for the conclusion that intracellular GlcNAc induces signaling in *C. albicans*. Although *ngt1Δ* cells were strongly defective in GlcNAc uptake, they still imported low levels of this sugar and grew slowly in the presence of high levels of GlcNAc [1]. It has been suggested that GlcNAc can be taken up inefficiently by other sugar transporters [30]. Analogous results were observed previously for *S. cerevisiae gal2* mutants that lack the galactose transporter [31]. The ability of *ngt1Δ* cells to take up low levels of GlcNAc was interesting because it correlated with the ability of *ngt1Δ* mutant cells to be stimulated to induce hyphal morphogenesis in the presence of GlcNAc at 1,000-fold higher levels than are required to induce the wild type [1]. This indicates that Ngt1 facilitates the uptake of GlcNAc into the cell, but that hyphal induction can occur without Ngt1 if cells are exposed to higher levels of GlcNAc. These data suggest that signaling is activated once a certain threshold level of intracellular GlcNAc is reached.

**Figure 1:**
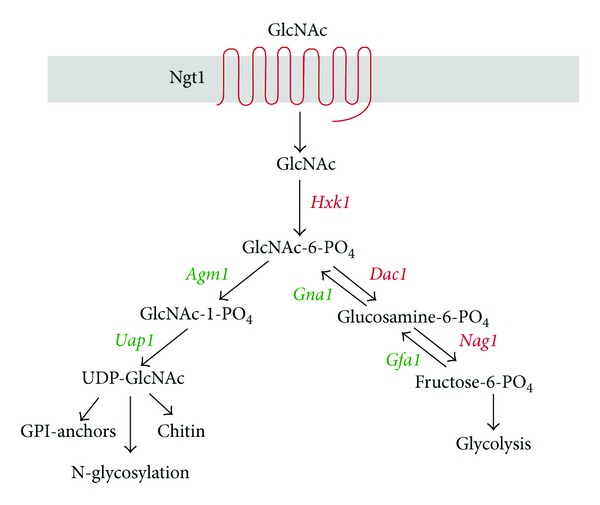
GlcNAc synthesis and degradation pathways in *C. albicans. *

The role of the intracellular GlcNAc was further defined by determining whether it had to be metabolized to induce signaling. Exogenous GlcNAc taken up by cells can enter the anabolic pathway to form UDP-GlcNAc, or it can be catabolized and used for energy. The role of GlcNAc catabolism was tested by mutating the* DAC1* and *NAG1* genes that encode the enzymes needed to deacetylate and then deaminate, GlcNAc-6-PO_4_, resulting in its conversion to fructose-6-PO_4_ (Figure 1). Interestingly, GlcNAc could still induce transcription of *NGT1* and other genes in the *nag1Δ* and *dac1Δ* mutants [2]. However, as discussed further below, GlcNAc inhibited the growth of these mutants so they could not be tested for hyphal morphogenesis. The anabolic pathway for synthesis of UDP-GlcNAc is essential and could not be tested easily. Therefore, to determine whether GlcNAc has to be metabolized to induce signaling, cells lacking the GlcNAc kinase gene, *HXK1*, were examined. GlcNAc has to be phosphorylated by Hxk1 to create GlcNAc-6-PO_4_ to proceed through either the anabolic or catabolic pathways (Figure 1). Significantly, an *hxk1Δ* mutant could still respond to GlcNAc to induce both the formation of hyphae and expression of *NGT1 *[2]. Similar results were obtained for a triple mutant lacking all three catabolic genes (*hxk1Δ nag1Δ dac1Δ*). These results indicate that GlcNAc metabolism is not required for signaling.

**Figure 2:**
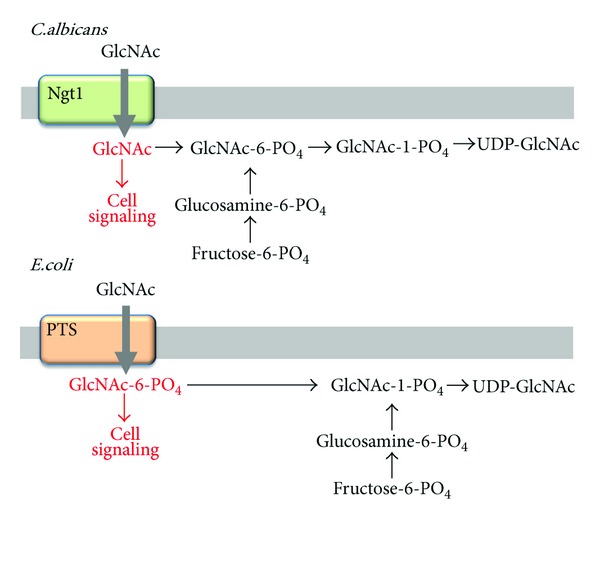
Comparison of GlcNAc synthesis pathways in fungi and bacteria. *C. albicans* and bacteria such as *E. coli* are thought to sense forms of GlcNAc (shown in red) that are not intermediates in the cellular hexosamine synthesis pathway. De novo synthesis pathways for GlcNAc are shown in black.

The ability of *hxk1Δ *and the *hxk1Δ nag1Δ dac1Δ* mutants, which lack the GlcNAc kinase, to respond to GlcNAc to undergo hyphal morphogenesis indicates that nonphosphorylated GlcNAc is capable of inducing *C. albicans*. Sensing the nonphosphorylated form of GlcNAc has important advantages for signal transduction. It allows cells to distinguish between the exogenous nonphosphorylated GlcNAc transported into the cell, and the GlcNAc-6-PO_4_ that is synthesized de novo within the cell (Figure 2). GlcNAc synthesis involves conversion of fructose-6-PO_4_ to glucosamine-6-PO_4_ and then to GlcNAc-6-PO_4_ [32]. Since nonphosphorylated GlcNAc is not synthesized by cells, it is not expected to be present intracellularly unless it is taken up from an exogenous source. Furthermore, detecting nonphosphorylated GlcNAc is also expected to allow cells to sense low levels of GlcNAc imported into the cell in the presence of high levels of endogenously synthesized GlcNAc-6-PO_4_. Large amounts of GlcNAc-6-PO_4_ are synthesized by cells for use in N-linked glycosylation, GPI anchors, and cell wall chitin. The capacity of cells to sense nonphosphorylated GlcNAc is also consistent with observations that glucosamine does not induce signaling in *C. albicans*, since glucosamine is expected to be converted to glucosamine-6-PO_4_ before it is converted to GlcNAc-6-PO_4_. The ability of *C. albicans* to sense intracellular nonphosphorylated GlcNAc therefore represents a novel signal transduction mechanism that may also occur in other organisms.

Sensing nonphosphorylated monosaccharides is not a unique strategy. Signaling pathways activated by other sugars have been reported to sense the nonphosphorylated form that is imported into the cell [33]. For example, galactose taken up by *S. cerevisiae* cells is bound by Gal3, which then promotes activation of the Gal4 transcription factor [34]. Also, it is common for metabolites to be direct mediators of cell signaling [35].

The subsequent pathways activated by GlcNAc are not known and are under investigation. One recent report suggested that GlcNAc might induce amino acid deprivation, which could be part of a signal to induce the response as morphological switch to hyphal formation [36].

### 3.3. Regulation of Gene Expression by GlcNAc

GlcNAc induces several different classes of genes, depending on the other environmental conditions and the cell type. These genes can be distinguished by whether they require a functional cAMP pathway to be induced by GlcNAc. For purposes of describing these gene sets in more detail below, the genes that are induced in a cAMP-independent manner will be referred to as GlcNAc induced. This set includes the genes that are needed for catabolism of GlcNAc, and also a group of genes involved in galactose catabolism, which are co-induced by GlcNAc for reasons that are unclear [22]. Two other sets of genes are induced by GlcNAc in a manner that requires a functional cAMP signal pathway. One set includes the genes that are induced during hyphal morphogenesis and another set includes the genes that are induced during White-Opaque switching, which is induced by GlcNAc in cells that are homozygous for the mating type locus [21]. Induction of these latter types of genes by GlcNAc is likely due to an indirect effect mediated by the cAMP pathway. The induction of specific genes during the hyphal or White-Opaque transitions also requires the other conditions in addition to GlcNAc, such as growth at specific temperatures (i.e., 37°C for hyphae and room temperature for White-Opaque switching). 

#### 3.3.1. GlcNAc-Induced Genes

**Figure 3:**
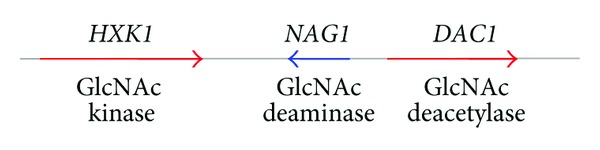
NAG regulon in *C. albicans*.The *C. albicans HXK1*, *NAG1*, and *DAC1* genes are clustered on chromosome 6 as shown in the figure.

After the initial discovery of the *HXK1*, *NAG1*, and *DAC1* genes that are needed for GlcNAc catabolism, characterization of their patterns of gene expression revealed that they are induced by GlcNAc [25, 26]. The *NAG1* and *DAC1* genes were identified in part based on their similarity to corresponding genes that promote the deacetylation and deamination of GlcNAc in bacteria [25, 26]. DNA sequence analysis revealed that the *NAG1* and *DAC1* genes are adjacent in the genome and that they are transcribed divergently. The *HXK1* gene was discovered fortuitously, since it is adjacent to *NAG1* (Figure 3). Thus, *HXK1*, *NAG1*, and *DAC1* genes are present in a cluster that was termed the NAG regulon [25].

Additional GlcNAc-induced genes have been identified in other studies. Analysis of the *HEX1* gene, which encodes a hexosaminidase that promotes the degradation of chitin oligomers (chitobiose), and the *NGT1* GlcNAc transporter, demonstrated that they are induced by GlcNAc [1, 37]. Genome wide microarray analysis of gene expression identified a novel GlcNAc-induced gene that was named *GIG1* [22]. Although its function is not clear, *GIG1* is highly conserved in fungi and may play a role in coordinating GlcNAc metabolism since its mutation confers resistance to Nikkomycin Z, which is thought to inhibit chitin synthase by mimicking UDP-GlcNAc. These other GlcNAc-induced genes are located at distinct sites in the genome and are not present in the NAG regulon.

GlcNAc also induces a subset of the galactose-regulated genes, which was unexpected since there is no obvious overlap between galactose and GlcNAc catabolism. *C. albicans* is not thought to synthesize galactose, and galactose does not induce GlcNAc catabolic genes [22]. However, galactose was detected in GlcNAc-induced *C. albicans* cells as part of a metabolomic study [36]. Further studies showed that GlcNAc catabolism was not required for *GAL10* to be induced in the *hxk1Δ* and the *hxk1Δ nag1Δ dac1Δ* mutants [2]. These data initially suggested that GlcNAc induction of *GAL* genes might be due to activation of Cph1, a transcription factor that promotes expression of both hyphal- and galactose-stimulated genes [38, 39]. The subset of *GAL *genes that were induced by GlcNAc was reported to be the only *C. albicans* genes that contain a putative Cph1 recognition motif in their upstream regulatory regions [38]. However, *cph1Δ* mutants could still be stimulated by GlcNAc to induce *GAL10* efficiently, so it remains unclear how the *GAL* genes are induced by GlcNAc [2]. Nonetheless, the regulation of the *GAL* genes may be physiologically significant. Recent studies indicate that the *GAL10* and *GAL102* gene are needed for cell wall integrity, even when grown in the presence of other sugars [40, 41].

Analysis of *C. albicans* cells grown in media with different sugars indicated that genes are directly induced by GlcNAc, not simply derepressed by absence of glucose [22]. Consistent with this, gel mobility shift assays showed that a new factor binds upstream of *NAG1* in GlcNAc-induced cells, suggesting these genes are activated rather than relieved of repression [25]. Surprisingly, GlcNAc did not repress the genes involved in synthesizing GlcNAc, *GFA1*, and *GNA1*. This is different from bacteria, as will be described below. Failure to detect repression of *GFA1* and *GNA1* expression in cells grown on GlcNAc medium indicates that GlcNAc synthesis is regulated by feedback inhibition, not by transcriptional regulation. Consistent with this, the first committed step in GlcNAc synthesis is known to be feedback inhibited by UDP-GlcNAc [32]. This step, which is carried out by Gfa1, involves a transfer of an amino group from glutamine to fructose-6-PO_4_ and subsequent isomerization to create glucosamine-6-PO_4_.

#### 3.3.2. Hyphal Genes

The genes that are induced during the switch to hyphal morphogenesis are typically referred to as hyphal genes, although most of these genes function in virulence and are not needed for hyphal growth [42]. The hyphal genes are regulated by many different factors in addition to GlcNAc, such as temperature, CO_2_, pH, nutrients, and peptidoglycan breakdown products in serum. The pathways activated by these different stimuli act in concert to regulate hyphal gene expression. The regulation of hyphal gene induction is a complex topic that has been reviewed recently [42–44]. Therefore, only a brief summary will be given here. The major pathway for stimulating hyphae appears to involve induction of adenylyl cyclase, which leads to activation of the cAMP pathway and stimulation of PKA kinases Tpk1 and Tpk2. Hyphal genes are then induced in a complex process that involves the transcription factor Efg1 being relieved of repression by Nrg1 [45].

#### 3.3.3. White-Opaque Genes

GlcNAc also stimulates *C. albicans* cells to undergo an epigenetic switch from the White phase morphology to the Opaque phase, which coincides with the change in expression of a large number of genes [21]. This switch is significant, as the White phase expresses genes that are predicted to make these cells better suited for systemic infections, whereas the Opaque cells express genes that should make them better suited for mucosal infections [46]. This switching pathway is also important because only the Opaque phase cells are competent for mating [46]. GlcNAc is thought to stimulate White-Opaque switching by activation of cAMP signaling and ultimately the Wor1 transcription factor, which appears to be a master regulator of switching [21, 47]. However, there are additional factors that control this switch [47]. Only *C. albicans* cells that are homozygous for mating type (a or *α*) are capable of being induced. Also, in vitro the switch to the Opaque phase occurs at room temperature whereas higher temperatures promote the switch back to the White phase. 

### 3.4. GlcNAc Induced Hyphal Morphogenesis

GlcNAc stimulates *C. albicans* budding cells to change their morphogenic program and instead form long filamentous cells with parallel cell walls that are termed hyphae [42]. The initial projection of polarized growth is usually referred to as a germ tube. As it elongates, nuclear replication occurs, and a septum is formed in the germ tube between the two nuclei. This multicellular structure is now termed a hypha, which continues to grow in an apical manner to produce new cell compartments. The highly polarized growth that forms a hypha is thought to be mediated by special structures at the apex that restrict growth to a narrow zone [48, 49]. The ability of *C. albicans* to undergo hyphal morphogenesis is thought to promote more aggressive invasive growth into tissues [50]. Interestingly, oral delivery of GlcNAc increased symptoms and fungal burden in a murine model of oral candidiasis [51]. It is not clear what regulates the morphological changes in cells after they switch from White to Opaque phase, so this section will only focus on hyphal morphogenesis.

GlcNAc stimulation of hyphal morphogenesis is thought to rely in part on the transcriptional changes described above [42]. So far, only one of the known hyphal-induced genes has been strongly implicated in promoting hyphal morphogenesis. This gene, *HGC1*, encodes a cyclin protein that complexes with and regulates the activity of the Cdc28 cyclin-dependent kinase [52]. As will be described in more detail below, Hgc1-Cdc28 phosphorylates key morphogenesis proteins to alter their activity in a manner that promotes polarized hyphal growth. The substrates for Hgc1-Cdc28 include the Rga2 protein that is a negative regulator of the cell polarity control protein Cdc42, the Sec2 protein that regulates the ability of Sec4 Rab GTPase to control delivery of secretory vesicles to sites of polarized morphogenesis, and the septin proteins that function in morphogenesis and cell septation [53]. Hgc1-Cdc28 also phosphorylates the transcription factor Efg1 to alter its ability to induce expression of genes involved in cell septation to help maintain long filamentous hyphal cells [54]. Interestingly, overexpression of *HGC1* is not sufficient to induce budding cells to undergo the transition to hyphal morphogenesis [52]. This suggests that GlcNAc may act in part by directly or indirectly activating Hgc1-Cdc28. 

The phosphorylation of Rga2 by Hgc1-Cdc28 prevents it from negatively regulating Cdc42 at hyphal tips [53]. Cdc42 is a Rho-type guanine nucleotide binding protein that when bound to GTP promotes polarized growth. During hyphal growth, Cdc42 localizes to the hyphal tip where it acts to restrict growth to this narrow zone. The Rga2 protein negatively regulates the activity of Cdc42 by acting as a GTPase activating protein (GAP) to promote hydrolysis of the GTP bound to Cdc42, thereby maintaining Cdc42 in an inactive GDP-bound state. However, phosphorylation of Rga2 by Hgc1-Cdc28 prevents Rga2 from localizing to hyphal tips [53]. This is thought to allow for continuous activation of Cdc42 at hyphal tips that mediates the highly polarized growth that forms the filamentous hyphal cells. 

Continued hyphal growth requires the delivery of secretory vesicles from the Golgi bodies to the site of growth marked by Cdc42. During transit from the Golgi bodies, secretory vesicles accumulate in an apical body called a Spitzenkörper, which appears to mediate the highly focused delivery of the secretory vesicles to the hyphal tip [49]. The transport of secretory vesicles from the Golgi bodies to the Spitzenkörper is regulated by a Rab family GTPase named Sec4, which is activated by a guanine nucleotide exchange factor (GEF) named Sec2. Interestingly, analysis of Sec2 showed that its localization to the Spitzenkörper depended on phosphorylation by the cyclin-dependent kinase Cdc28 complexed with either the Hgc1 or Ccn1 cyclins [55]. Failure to localize Sec2 to the Spitzenkörper prevented normal hyphal growth.

Septin proteins are also regulated by phosphorylation by the Hgc1-Cdc28 kinase. The septins are a family of GTP-binding proteins that are distinct because they also form filaments at the cortex of the plasma membrane in fungi [56, 57]. The septins were initially discovered for their role in budding yeast cell septation, but are now known to participate in other types of polarized morphogenesis including hyphal growth of *C. albicans* [58, 59]. Septin function during hyphal growth is regulated in part by phosphorylation [60]. Hgc1-Cdc28 is required to maintain phosphorylation of the Cdc11 septin on Ser95 during hyphal growth, and this promotes the continued polarized morphogenesis that forms the elongated hyphal cells. Hgc1-Cdc28 also contributes to the phosphorylation of the septin Sep7, which is thought to prevent activation of the cytokinesis pathways so that the hyphal cells persist as long chains of filamentous cells, rather than separating as occurs during growth by budding [61]. 

An additional mechanism that prevents hyphal cells from undergoing cytokinesis and separating into individual cell units is mediated by Hgc1-Cdc28. This cyclin-CDK complex phosphorylates the Efg1 transcription factor, which prevents Efg1 from activating the expression of the genes that encode the enzymes responsible for degrading the cell wall material between neighboring cells following septation [54]. Phosphorylated Efg1 is thought to bind to the promoters of these genes and prevent their expression. 

### 3.5. GlcNAc Inhibits Growth of nag1 and dac1 Mutants

Studies on the *C. albicans* mutants that are defective in catabolizing GlcNAc revealed the surprising finding that GlcNAc can inhibit the growth of a subset of these mutants [2]. The *nag1Δ* and *dac1Δ* mutants that fail to deacetylate and deaminate GlcNAc, respectively, quickly ceased growth in the presence of GlcNAc, even when another sugar was available for nutrition. For example, growth rate studies in liquid showed that addition of 0.1 mM GlcNAc was sufficient to rapidly inhibit growth of the *nag1Δ*  and *dac1Δ* mutants even though 50 mM galactose was present as a nutrient source [2]. Although growth was blocked, the *nag1Δ* and *dac1Δ* mutants maintained viability in the presence of GlcNAc, indicating GlcNAc was inhibitory but not lethal. The observation that growth of the *nag1Δ* and *dac1Δ* mutants was inhibited by GlcNAc, but not the growth of the *hxk1*Δ mutant that fails to phosphorylate GlcNAc or the triple *hxk1Δ nag1Δ dac1Δ* mutant, suggested that an excess of GlcNAc-6-PO_4_ is toxic to cells. Similar deleterious effects of GlcNAc on growth were observed for *E. coli* mutants that lack GlcNAc deacetylase or deaminase activity that were also attributed to excess GlcNAc-6-PO_4_ [62]. In *E. coli*, growth was quickly restored by adding exogenous uridine [10], suggesting that conversion of excess GlcNAc-6-PO_4_ to UDP-GlcNAc depleted UTP levels. However, addition of excess uridine to the medium did not strongly rescue the *C. albicans nag1Δ* and *dac1Δ* mutants from growth inhibition by GlcNAc [2]. A similar type of growth inhibition was reported for the* gndΔ* mutant of the protozoan *Leishmania*, which lacks an ortholog of the *NAG1* glucosamine deaminase gene [11]. However, in this case the inhibitory effect of GlcNAc on the *Leishmania gndΔ* mutant appears to be distinct from that seen for the *C. albicans nag1Δ* and *dac1Δ* mutants. The inhibition of the *Leishmania gndΔ* mutant could be overcome by adding alternative carbon sources, such as glycerol and was concluded to be due to depletion of ATP [11].

An interesting speculation is that an inhibitory effect of GlcNAc on growth, similar to that observed for the *C. albicans nag1Δ* and *dac1Δ* mutants, could have played a role in loss of the GlcNAc catabolic genes in *S. cerevisiae, S. pombe*, and related species. The *HXK1, NAG1,* and *DAC1* genes are clustered together in *C. albicans *(Figure 3), an arrangement that is also conserved in other species [25, 26, 63]. The clustering of these genes might facilitate their simultaneous loss, which would be beneficial in the event that a spontaneous *nag1* or *dac1* mutant was exposed to GlcNAc. Instead of remaining inhibited, deletion of the cluster, including the *HXK1* GlcNAc kinase gene, would relieve cells of the inhibitory effects of GlcNAc on growth.

## 4. GlcNAc Roles in Multicellular Organisms

### 4.1. Overview of GlcNAc Signaling Roles in Multicellular Organisms

The ability of multicellular organisms to respond directly to exogenous GlcNAc has not been well studied. One reported response of mammalian cells to exogenous GlcNAc is to alter cell-signaling pathways by changing the patterns of N-linked glycosylation of plasma membrane proteins [7]. There is also a very rapidly growing body of work demonstrating that multicellular organisms, and possibly some filamentous fungi, use O-linked attachment of GlcNAc as an important posttranslational modification on intracellular proteins [64]. This modification is mediated by the enzyme O-GlcNAc transferase (OGT), which catalyzes the transfer of a GlcNAc moiety from UDP-GlcNAc to Ser or Thr residues on cytoplasmic and nuclear proteins. As will be described further below, a large number of substrates have been identified for OGT indicating that this modification broadly regulates many aspects of cellular function [6]. Another modification is the attachment of GlcNAc to fucose residues on the extracellular side of the Notch family proteins [8]. This modification is carried out by the Fringe protein and has the effect of modulating signaling activity by altering the ligand specificity of Notch. These forms of posttranslational modification have been reviewed recently, so they will only be summarized below.

### 4.2. GlcNAc-Induced Changes in N-glycans

Addition of exogenous GlcNAc to mammalian cells has been shown to alter signal transduction pathways [7, 65]. For example, GlcNAc can inhibit T cell responsiveness and it can also alter IL-3 receptor-*α* expression at the cell surface [66, 67]. GlcNAc is thought to cause these effects by increasing the production of branched N-glycans on cell-surface proteins. Changes in N-glycan branching can have profound effects on cell surface protein organization and is thought to underlie many aspects of normal metabolic responses as well as dysregulated function in diseases such as autoimmunity, cancer, and metabolic syndromes [7].

In one recent study, exogenous GlcNAc added to mammalian cells appeared to be taken up by pinocytosis and was then converted to UDP-GlcNAc [68]. Very little GlcNAc, if any, appeared to be catabolized [67]. It will be interesting to determine if this is also true for a broader range of cell types, since mammalian genomes encode orthologs of the GlcNAc catabolic enzymes. The rise in the intracellular pool of UDP-GlcNAc caused by the uptake of exogenous GlcNAc is thought to lead to increased branching of the N-linked carbohydrate chains that are added to proteins in the Golgi bodies. Part of the evidence to support this is that the effects of GlcNAc can be reversed by inhibitors that block N-glycan branching [68], and the effects of GlcNAc can be mimicked by overproducing the Mgat5 N-acetylglucosaminyltransferase that promotes N-glycan branching [67]. It remains to be examined whether O-linked GlcNAc is also altered when cells are exposed to exogenous GlcNAc, and whether this modification contributes to the effects that are observed after treating mammalian cells with GlcNAc.

### 4.3. O-GlcNAc Modification of Intracellular Proteins

O-GlcNAc modification of proteins is thought to occur in a wide range of eukaryotic cells, although it has been most extensively studied in animal cells [6]. It occurs when OGT transfers the GlcNAc moiety of UDP-GlcNAc onto the Ser and Thr residues of proteins. The reaction is reversible; there is another enzyme, O-GlcNAcase (OGA), that is capable of removing the GlcNAc. These reactions can occur in the cytoplasm or the nucleus. Although initially it was difficult to identify O-GlcNAc modified proteins, new methods have permitted the identification of a large number of proteins, probably more than 1,000 O-GlcNAc modified proteins [6, 9]. O-linked GlcNAc is found on a diverse array of proteins that are involved in many different aspects of cellular function, including transcriptional regulation, splicing, translation, signal transduction, cell cycle progression, mitochondrial metabolism, protein degradation by the proteosome, and others. Recent studies have also linked O-GlcNAc modification of chromatin proteins to the regulation of epigenetic changes in gene expression [9].

OGT activity appears to be regulated by the available levels of its substrate, UDP-GlcNAc [9]. The fact that de novo synthesis of UDP-GlcNAc involves a sugar (fructose-6-PO_4_), an amino acid (glutamine), acetate from Acetyl CoA, and a nucleotide (UTP) has led to the suggestion that O-GlcNAc modification represents a sensor for the status of diverse nutrients and metabolites. Because of this link to nutritional status, O-GlcNAc has been implicated in a range of human diseases, including diabetes and cancer [6]. 

Identification of the sites of O-GlcNAc attachment has revealed that some proteins are modified by phosphorylation on the same residues to which O-GlcNAc is attached [6]. These modifications are exclusive, so only one or the other will occur. This indicates that O-GlcNAc competes with protein kinase pathways for regulation of cellular proteins. Furthermore, this discovery indicates that studies aimed at defining the functional roles of specific phosphorylation sites by site-directed mutagenesis of Ser and Thr residues may lead to erroneous conclusions. In view of the widespread occurrence of O-GlcNAc on proteins, mutant phenotypes observed after mutation of Ser or Thr residues can no longer be assumed to be due only to the absence of phosphorylation, as O-GlcNAcylation may also be affected.

Emerging studies on other organisms have found additional roles for O-GlcNAc. Plants contain two OGTs, termed spindly and secret agent, that act as negative regulators of the gibberellin signaling pathway [69]. They are also important for sensing environmental signals, circadian rhythms, development, intercellular transport, and virus infection [70]. Although OGT genes are present in some fungi, little is known about their function since OGT is absent from the commonly studies model yeasts, *Saccharomyces cerevisiae* and *Schizzosaccharomyces pombe*. Interestingly, higher plants appear to lack the OGA enzyme that removes GlcNAc [70].

### 4.4. GlcNAc Modification of Fucose Residues on Extracellular Signaling Proteins

The Notch family of receptor proteins responds to ligands at the cell surface. Signaling through these receptors plays key roles in determining cell fate during development [71]. Abnormal signaling activity by the Notch family of receptors has also been implicated in human diseases, such as cancer [72]. Interestingly, studies on the structure and function of Notch have revealed that O-linked attachment of fucose glycans onto the Notch extracellular domain modulates the signaling activity of Notch [8]. In particular, these sugars are added to the epidermal-growth-factor (EGF-) like repeats in the extracellular domain of Notch. Furthermore, Fringe, genetically known to be a known modifier of Notch function, is an O-fucose specific *β*-(1,3)-N-acetylglucosaminyltransferase that modifies the fucose residues on Notch. The attachment of GlcNAc onto O-fucose on Notch by Fringe improves signaling by the Delta class of Notch ligands, but inhibits signaling by the Serrate class of Notch ligands [8]. Additional carbohydrate modifications of Notch further regulate its activity, providing new paradigms for the regulation of cell signaling by glycosylation. 

## 5. Bacterial GlcNAc Pathways

A major role for GlcNAc signaling in bacterial cells is to coordinate the de novo synthesis of GlcNAc with the recycling or catabolism of GlcNAc released during cell wall remodeling. This coordinate regulation is designed to ensure an appropriate supply of UDP-GlcNAc for new cell growth. Interestingly, the GlcNAc sensing proteins that regulate GlcNAc synthesis and catabolism also regulate other important pathways. For example, GlcNAc regulates virulence properties of pathogenic bacteria, such as the production of fimbriae and CURLI fibers in *E. coli* [5, 73]. Exogenous levels of GlcNAc also regulate the developmental switch to sporulation and antibiotic production in soil bacteria [74]. Recent studies have also shown that O-GlcNAc modification occurs in bacteria [75]. These results demonstrate that cells use GlcNAc as a signaling molecule in addition to simply regulating metabolic pathways.

### 5.1. Regulation of GlcNAc Synthesis in *E. coli*

**Figure 4:**
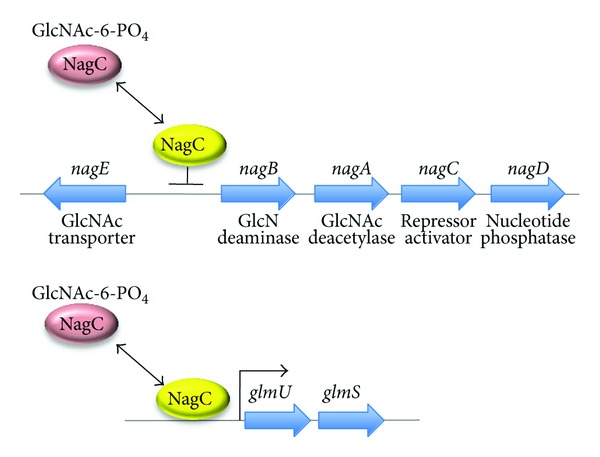
Regulation of GlcNAc synthesis and catabolic genes in *E. coli. *The upper image illustrates the regulation of the GlcNAc catabolic genes and the lower panel shows the GlcNAc synthesis genes. Note that the NagC transcriptional regulator acts as a repressor of the *nagABCDE* operon but is an activator of the *glmUS* operon. Binding of GlcNAc-6-PO_4_ alters NagC so that the expression of the GlcNAc catabolic genes (*nagABCDE)* is increased and the expression of the synthesis genes (*glmUS*) is decreased. (Note: gene sizes are not drawn to scale in order to present NagC regulation more clearly.)

The regulation of GlcNAc metabolism has been well studied in *E. coli* and will be reviewed here as an example of how GlcNAc pathways are controlled in bacteria. Transcriptional regulation of GlcNAc synthesis, recycling, and catabolism are interrelated processes in *E. coli*. The GlcNAc synthesis genes *glmU* and *glmS* are present in one operon, and the GlcNAc catabolic genes are located in a divergent operon that contains the *nagEBACD* genes [76] (Figure 4). Both operons are regulated by the transcription factor NagC, but in an opposite manner [77, 78]. NagC represses the *nagEBACD* operon to prevent expression of the GlcNAc catabolic genes; binding of GlcNAc-6-PO_4_ to NagC causes allosteric changes that derepress its repressor function and thereby promotes the expression of the genes needed for GlcNAc catabolism. By contrast, NagC acts as an activator at the *glmUS* operon. In the absence of GlcNAc-6-PO_4_, NagC binds upstream and activates expression of the *glmUS* operon to promote GlcNAc synthesis [4, 78]. Bacteria are distinct from eukaryotic cells in that they do not synthesize GlcNAc-6-PO_4_ (Figure 3 and [32]). They instead convert glucosamine-6-PO_4_ directly to GlcNAc-1-PO_4_. Intracellular GlcNAc-6-PO_4_ is therefore expected to occur in bacteria only as a result of the import of exogenous GlcNAc (Figure 3). Thus, bacteria can distinguish exogenous GlcNAc from the forms of GlcNAc synthesized de novo in the cell in a manner that is analogous to the detection of nonphosphorylated GlcNAc in *C. albicans*. 

Synthesis of UDP-GlcNAc starts with fructose-6-PO_4_ being converted to glucosamine-6-PO_4_ by GlmS [79]. This enzyme transfers an amino group from glutamine to the 2 position of fructose-6-PO_4_ to create glucosamine-6-PO_4_. A phosphoglucosamine mutase (GlmM) converts it to glucosamine-1-PO_4_ [80]. The final steps are carried out by a protein with two functional domains (GlmU); one domain acts as a glucosamine-1-PO_4_ acetyltransferase and the other as a GlcNAc-1-PO_4_ uridyltransferase [81]. Thus, although generally similar to eukaryotes, bacterial synthesis of UDP-GlcNAc is distinct.

The synthesis of glucosamine-6-PO_4_ by GlmS is the rate-limiting step in GlcNAc synthesis, so it is not surprising that it is under special forms of regulation. One interesting type of feedback regulation mediated by glucosamine-6-PO_4_ involves GlmZ, a small RNA (sRNA) [82]. The primary glmUS transcripts are processed by RNAse E at the glmU stop codon [83]. Upon a decrease in the intracellular glucosamine-6-PO_4_ concentration, the *glmS* monocistronic transcript is stabilized in a process that depends on GlmZ, a sRNA encoded at a distal site in the genome. The base-pairing of GlmZ to a *∼*15 nucleotide single-stranded stretch releases the sequestered *glmS* Shine-Dalgarno sequence to increase translation, which also strongly stabilizes the glmS mRNA [83, 84]. Additional components of this regulatory pathway involve YhbJ and the sRNA GlmY, which control processing and stability of GlmZ. The sRNA GlmY appears promote accumulation of active full-length GlmZ by acting as a molecular mimic and interfering with the processing of GlmZ to a shorter inactive form [82, 84]. Note that the synthesis of GlmU and GlmS is strictly coupled at the transcriptional level. GlmU is always required, since it turns over both externally and internally derived substrates, whereas GlmS is only essential for synthesis of glucosamine-6-PO_4_ in the absence of external amino sugars. Thus, GlmZ and GlmY act to specifically control the level of GlmS at the posttranscriptional level.

Another remarkable example of GlmS regulation occurs in Gram-positive bacteria. Glucosamine-6-PO_4_ acts as a cofactor for the ribozyme activity of the *glmS* mRNA, which results in its cleavage [85]. Since *glmS* encodes a glucosamine-6-PO_4_ synthase, this mechanism coordinates expression of glmS with the intracellular concentration of its product, glucosamine-6-PO_4_. Thus, there are at least two mechanisms by which different bacteria regulate expression of GlcNAc synthesis genes in the presence of exogenous GlcNAc. These mechanisms act to keep the flux through the hexosamine pathway constant even when environmental and nutritional conditions require a change in peptidoglycan and LPS synthesis rates. 

### 5.2. GlcNAc Recycling and Catabolism

Peptidoglycan, also known as murein, is the rigid shape-forming layer of the bacterial cell wall. Peptidoglycan undergoes extensive degradation and resynthesis during active growth. It is estimated that ~50% of the sidewall peptidoglycan is broken down and reused each generation [13]. The principal degradation product is the anhydro-muropeptide GlcNAc-*β*-1,4-anhydro-N-acetylmuramyl (anhMurNAc)-L-alanyl-*γ*-D-glutamyl-meso-diaminopimelyl-D-alanine [13]. Muropeptides are imported into the cytoplasm via a specific permease, AmpG. The NagZ protein functions as a *β*-N-acetylglucosaminidase to release the nonphosphorylated GlcNAc moiety, which is then phosphorylated to GlcNAc-6-PO_4_ by the NagK kinase [86]. The GlcNAc-6-PO_4_ appears to be deacetylated by NagA and reused to synthesize UDP-GlcNAc as described above. There may also be an alternate pathway that has not yet been defined [86]. 

Some GlcNAc resulting from peptidoglycan turnover is also imported into the cell through the NagE phosphotransferase system that transports exogenous GlcNAc across the inner membrane [87]. The GlcNAc transported into the cytoplasm by NagE is immediately phosphorylated by the phosphotransferase system to form GlcNAc-6-PO_4_ [88, 89]. The GlcNAc-6-PO_4_ can then be recycled after its deacetylation to Glucosamine-6-PO_4_ by NagA and conversion to UDP-GlcNAc by the action of GlmM and GlmU, as described above. Alternatively, glucosamine-6-PO_4_ can enter the glycolytic pathway after it is deaminated by NagB and isomerized to fructose-6-PO_4_.

Additional genes have been found to be under control of NagC and are thus regulated by GlcNAc. For example, NagC represses the *chbBCARFG* operon in the absence of GlcNAc. This operon encodes proteins involved in uptake and degradation of chitobiose, a *β*-(1,4)-linked dimer of GlcNAc, which is present in the environment as a result of the breakdown of chitin by chitinases [90]. The *chb* operon is also repressed by the dual function regulator ChbR [91]. In the absence of chitobiose, both NagC and ChbR act as repressors. However, both GlcNAc and chitobiose are required to activate *chb* expression. This dual regulation, one signal for NagC to relieve its repression and another signal for ChbR to activate the expression of the *chb* operon, ensures that this operon is only induced in the presence of chitobiose. NagC is also involved in the repression of the galactose transporter *galP* [92]. It is unclear what biological advantage this provides, but it is potentially interesting since GlcNAc induces the *GAL* genes in *C. albicans* [22]. 

### 5.3. GlcNAc Regulation of Virulence Properties

Some of the virulence factors of *E. coli* are regulated by GlcNAc, such as fimbriae and CURLI fibers, as will be described below. GlcNAc may also have other roles in virulence, since the ability of *E. coli* to catabolize GlcNAc is also important for it to colonize the G.I. tract [93].

#### 5.3.1. Fimbriae

Bacterial attachment to host cells plays a central role in colonization and is often crucial in pathogenesis. *E. coli* produces a variety of fimbrial adhesins that permit attachment to specific host receptors. Type 1 fimbriae have been identified as a virulence factor in urinary tract infections [94]. This adhesin promotes invasion of uroepithelial cells and may also contribute to chronic inflammatory diseases, such as Crohn's and interstitial cystitis. Interestingly, production of type 1 fimbriae is inhibited by GlcNAc [5]. The GlcNAc is thought to be released from the extracellular matrix by the action of the host defenses mechanisms [5]. Sialic acid, which is broken down to GlcNAc-6-PO_4_ by catabolism in *E. coli*, can also inhibit production of fimbriae [95]. In addition to acting as adhesins, type 1 fimbriae are significant to the progression of infection because they are proinflammatory. Thus, inhibition of type 1 fimbriation by GlcNAc appears to modulate the interaction between *E. coli* and the host in ways that will promote dissemination of an infection.

Production of type 1 fimbriae in *E. coli* is regulated by a process termed phase variation in which the expression of the *fim* genes is switched on or off. Phase variation is due to a DNA inversion catalyzed by FimB (which promotes switching in either direction) or FimE (mainly on-to-off switching). Blocking FimB action will therefore generate afimbriate cells. GlcNAc was found to inhibit type 1 fimbriae production by preventing NagC, the GlcNAc-6P-responsive repressor of GlcNAc catabolism, from acting in a positive manner to promote expression of *fimB*. [5]. In addition to inhibiting *fimB* expression, addition of GlcNAc was also found to inhibit FimB from promoting DNA inversion (3- and >35-fold, resp.) [5]. The authors suggested that since GlcNAc levels rise during inflammation, this could signal to the bacterium that host defenses are activated. Inhibiting the expression of fimbrial adhesins, which are proinflammatory, would therefore help limit inflammation.

#### 5.3.2. CURLI Fibers

CURLI fibers are extracellular molecules that important for pathogenesis because of their role in biofilm formation, adhesion, and the internalization of* E. coli *by epithelial cells [73]. Production of CURLI fibers, like type 1 fimbriae, is reduced by GlcNAc [96]. The mechanism by which GlcNAc inhibits production of CURLI fibers is not known. However, it is interesting that production of CURLI is also decreased in strains that are undergoing a high rate of cell wall lysis, possibly due to GlcNAc derived from peptidoglycan degradation [97]. As described above for fimbriae, the decreased production of CURLI fibers after exposure of cells to GlcNAc may be a mechanism to balance the interaction between *E. coli* and the host immune response, since CURLI are also proinflammatory. Decreased production of CURLI fibers may also influence the progression of an infection by promoting the dissemination of bacteria within the host, since these molecules promote biofilm formation and adhesion. 

### 5.4. O-GlcNAc Modification of Flagella Proteins

O-GlcNAc modification of proteins has also been identified in bacteria and appears to be carried out by O-GlcNAc transferases and O-GlcNAcases similar to those in multicellular organisms [75, 98, 99]. Interestingly, O-GlcNAc has been linked to the regulation of cell motility in the pathogenic bacteria *Listeria monocytogenes* [75]. Motility is mediated by flagella, and the FlaA subunits of* L. monocytogenes* flagella were found to be covalently modified by O-linked GlcNAc at three to six sites per subunit [98]. The glycosylation sites are all located in the central region of the FlaA protein that is exposed to the surface. Subsequent studies showed that GmaR is the O-GlcNAc transferase that catalyzes the O-GlcNAc modification of FlaA [75]. These studies identified GmaR as the first O-GlcNAc transferase in bacteria. Subsequent studies identified O-GlcNAcases in bacteria [99].

Although GmaR catalyzes O-GlcNAc modification of flagella and is required for bacterial motility, the function of O-GlcNAc modification of FlaA is unclear [75]. One reason for this is that *gmaR* mutants display a defect in flagellar gene transcription. The *gmaR* mutant phenotype is complex because GmaR is a multifunctional protein that also promotes expression of flagellar genes. GmaR regulates flagellar gene expression by binding directly to MogR and inhibiting its ability to bind target sequences in promoters of the flagellar genes to repress their expression. This anti-repressor function of GmaR is independent of its O-GlcNAc transferase function. Thus, GmaR is unique in that it is an O-GlcNAc transferase that also transcriptionally regulates the expression of its substrate.

Although the effects of O-GlcNAc modification of FlaA remain to be determined, it is likely to contribute to flagellar regulation. Flagellar motility is essential for bacteria to acquire nutrients, colonize surfaces, and establish infections [100]. However, it is also important for bacteria to properly regulate flagella production and motility. For example, although flagella enhance adherence and invasion in the early stages of host infection, continuous production of flagella during infection stimulates innate immune responses [101–103]. To avoid this, many bacterial pathogens turn off production of flagella after initiating an infection. In the case of *L. monocytogenes,* the transcription of flagellar motility genes is repressed at human body temperature. 

### 5.5. GlcNAc Regulation of Morphogenesis and Antibiotic Production in Soil Bacteria

GlcNAc regulates the ability of soil bacteria to undergo sporulation and to induce the production of antimicrobial compounds. Studies on *Streptomyces coelicolor* have shown that nutrients in the soil influence the decision by this filamentous bacterium to continue vegetative growth under good conditions, or switch to a new morphogenetic program under adverse conditions that leads to production aerial hyphae and stress-resistant spores [104]. GlcNAc has specific effects on this decision, since other comparable carbon and nitrogen sources do not cause apparent effects on development [105]. 

Analysis of the regulation of *S. coelicolor* morphogenesis by GlcNAc led to a “feast or famine” model [74]. Addition of GlcNAc to cells grown in rich media (feast condition) promoted growth and prevented sporulation, whereas, exposure to GlcNAc under poor nutritional conditions (famine condition) promoted the developmental transition leading cells to sporulate. It was suggested that during famine conditions the availability of GlcNAc is likely due to cell wall hydrolysis, which would trigger the development of spores that are more resistant to a harsh environment. In contrast, the availability of GlcNAc during feast conditions may be derived from other sources, such as fungal chitin, and would block development to permit continued vegetative growth [74]. 

GlcNAc must be taken up by the cells to have an effect; mutation of the phosphotransferase system that appears to account all of the transport of GlcNAc into *S. coelicolor* prevented the effects of GlcNAc [106]. Mutational studies also indicated that intracellular GlcNAc is converted into glucosamine-6-PO_4_, which then binds to the repressor DasR and leads to derepression of genes involved in GlcNAc catabolism and antibiotic production [74, 104]. Thus, DasR acts in a manner analogous to NagC in *E coli*, although DasR appears to respond to glucosamine-6-PO_4_ rather than GlcNAc-6-PO_4_. The mechanism by which GlcNAc promotes sporulation under famine conditions, and prevents it under feast conditions, is thought to involve complex interplay between metabolic and developmental pathways [107, 108].

GlcNAc was also found to regulate the production of secondary metabolites that have antimicrobial activity by *S. coelicolor* and other *Streptomyces* species [74]. Production of these compounds is stimulated by GlcNAc under famine conditions. This gives added significance to the analysis of GlcNAc regulation in *Streptomyces*, since it has biotechnology applications for improving antibiotic production. *Streptomycetes* are well known for their ability to produce metabolites with interesting properties, including about 50% of all known antibiotics as well as other compounds with anticancer antifungal, and immunosuppressant activity [109].

Another example of the regulation of secondary metabolite production by GlcNAc occurs in *Pseudomonas aeruginosa*. This opportunistic pathogen was recently reported to respond to GlcNAc in the lung secretions (sputum) of Cystic Fibrosis patients [110]. Microarray analysis revealed that growth of *P. aeruginosa* in sputum induced expression of the GlcNAc catabolic genes, consistent with the presence in sputum of GlcNAc-containing polymers, such as hyaluronic acid and mucins [110]. Interestingly, GlcNAc also induced the production of phenazine antimicrobial compounds by *P. aeruginosa*. It was proposed that this antimicrobial defense system might be induced by GlcNAc since *P. aeruginosa* also lives in the soil where GlcNAc would be an indicator of the presence of other bacteria or fungi in the area.

## 6. Interspecies Communication

 In recent years there has been growing awareness that chemical messengers are not only used to communicate in an intraspecies fashion, they can also act between species [111–113]. Some quorum sensing molecules and hormones have been found to transduce signals between species, and even across kingdoms. In other cases, the synthesis of secondary metabolites contributes to interspecies signaling. The presence of GlcNAc on the surface of so many different cell types, combined with the fact that cells in nature typically grow in mixed microbial environments, makes GlcNAc well suited to be part of the communication that goes on between cells. In this regard, it is interesting that epithelial cells and macrophages produce chitinase even though humans do not produce chitin, indicating that the chitinase is targeted for destroying invading species and possibly activating cell signaling pathways as well [114].

One example of interspecies communication that was described above is that inflammation due to bacterial infection can result in increased release of amino sugars from mammalian host cells, which in turn can signal *E. coli* to decrease production of type 1 fimbriae and CURLI fibers [5, 96]. Decreasing the production of these proinflammatory cell surface adhesion molecules would reduce the level of inflammation and also help to disseminate the infection.

Emerging data also indicate that there are interesting forms of interspecies communication between *C. albicans* and *P. aeruginosa*. *C. albicans* undergoes opposing morphological transitions in response to bacterial cell wall breakdown products, including GlcNAc, and a quorum factor produced by *P. aeruginosa* (3-oxo-C12 homoserinelactone). GlcNAc and other peptidoglycan breakdown products, such as muramyl dipeptide, induce *C. albicans* to undergo hyphal morphogenesis [18, 115], whereas the quorum factor 3-oxo-C12 homoserinelactone promotes the budding pattern of growth [116]. This modulation of* C. albicans* morphogenesis is also significant since *P. aeruginosa* can adhere to the filamentous hyphal cells and form a dense biofilm that kills the *C. albicans* cells [117]. Conversely, the *C. albicans* budding yeast cells that are induced by the quorum factor 3-oxo-C12 homoserinelactone are protected from being killed, since they are not attacked by *P. aeruginosa* [116]. The interspecies communication is further enhanced by the ability of *P. aeruginosa* cells to be stimulated by farnesol, a quorum factor produced by *C. albicans*. The farnesol produced by *C. albicans* accumulates in the medium where it interferes with quorum signaling in *P. aeruginosa* and prevents induction of virulence factors [118]. Thus, GlcNAc forms one part of a complex interspecies exchange of signaling molecules between *C. albicans* and *P. aeruginosa*. Given the widespread occurrence of GlcNAc at the surface of diverse organisms, it is likely that future studies will reveal new roles for GlcNAc in other forms of interspecies communication that promote either helpful symbiotic relationships or pathogenic interactions. 

## 7. Concluding Comments

 The ubiquitous presence of GlcNAc at the surface of diverse cell types from bacteria to humans provides opportunities for this amino sugar to participate in cell signaling, analogous to other common types of molecules that act as messengers including ions, nucleotides, and amino acids. The recent advances in identifying novel roles for GlcNAc in cell signaling indicate that future studies will continue to map out additional roles for GlcNAc in an even broader range of cell types. In particular, it will be interesting to define the roles for GlcNAc signaling in complex ecological niches, such as the human gut, where a broad range of bacteria, fungi, and human cells coexist. The microbes in the human gut must be balanced between beneficial effects for the host and pathogenic effects when the microbiome is dysregulated [119–121]. In this regard it is also interesting that GlcNAc was reported to have beneficial therapeutic effects on patients with inflammatory bowel disease, suggesting that new studies in this area could lead to novel therapies for human disease [122].
